# Exploration of Lipid Metabolism in Relation with Plasma Membrane Properties of Duchenne Muscular Dystrophy Cells: Influence of L-Carnitine

**DOI:** 10.1371/journal.pone.0049346

**Published:** 2012-11-27

**Authors:** Françoise Le Borgne, Stéphane Guyot, Morgan Logerot, Laurent Beney, Patrick Gervais, Jean Demarquoy

**Affiliations:** 1 Laboratoire Bio-PeroxIL, Biochimie du Peroxysome, Inflammation et Métabolisme Lipidique, Université de Bourgogne - Faculté des Sciences Gabriel, Dijon, France; 2 UMR A 02.102 Procédés Alimentaires et Microbiologiques, Equipe Procédés Microbiologiques et Biotechnologiques, AgroSup Dijon/Université de Bourgogne, bât Erasme, Dijon, France; Mayo Clinic, United States of America

## Abstract

Duchenne muscular dystrophy (DMD) arises as a consequence of mutations in the dystrophin gene. Dystrophin is a membrane-spanning protein that connects the cytoskeleton and the basal lamina. The most distinctive features of DMD are a progressive muscular dystrophy, a myofiber degeneration with fibrosis and metabolic alterations such as fatty infiltration, however, little is known on lipid metabolism changes arising in Duchenne patient cells. Our goal was to identify metabolic changes occurring in Duchenne patient cells especially in terms of L-carnitine homeostasis, fatty acid metabolism both at the mitochondrial and peroxisomal level and the consequences on the membrane structure and function. In this paper, we compared the structural and functional characteristics of DMD patient and control cells. Using radiolabeled L-carnitine, we found, in patient muscle cells, a marked decrease in the uptake and the intracellular level of L-carnitine. Associated with this change, a decrease in the mitochondrial metabolism can be seen from the analysis of mRNA encoding for mitochondrial proteins. Probably, associated with these changes in fatty acid metabolism, alterations in the lipid composition of the cells were identified: with an increase in poly unsaturated fatty acids and a decrease in medium chain fatty acids, mono unsaturated fatty acids and in cholesterol contents. Functionally, the membrane of cells lacking dystrophin appeared to be less fluid, as determined at 37°C by fluorescence anisotropy. These changes may, at least in part, be responsible for changes in the phospholipids and cholesterol profile in cell membranes and ultimately may reduce the fluidity of the membrane. A supplementation with L-carnitine partly restored the fatty acid profile by increasing saturated fatty acid content and decreasing the amounts of MUFA, PUFA, VLCFA. L-carnitine supplementation also restored muscle membrane fluidity. This suggests that regulating lipid metabolism in DMD cells may improve the function of cells lacking dystrophin.

## Introduction

In Duchenne muscular dystrophy patient, muscle cells are lacking dystrophin. Dystrophin functions as part of a large protein complex that includes dystroglycans, sarcoglycans, dystrobrevins, syntrophins, and sarcospan [1]. The absence of dystrophin has a dramatic impact on cell membrane stability and structure as this dystrophin–glycoprotein complex mechanically stabilizes the sarcolemma against shear stresses imposed during muscle activity [2]. In the early stages of the disease, muscle undergoes active regeneration [3], [4], [5], [6], but as the disease progresses, the regeneration process is not efficient enough and muscle degeneration exceeds the regeneration process leading to muscle loss.

To cure DMD, gene therapy is likely to be the only efficient approach, but its application will still need many years before being routinely used in patients [7], [8]. In the interval, the development of palliative treatments appears very useful [9].

L-carnitine is a small molecule derived from lysine and methionine. It is implicated in the fatty acid metabolism both at the mitochondrial and peroxisomal levels [10] and as a cofactor in several other cellular functions such as the acetylation of proteins. L-carnitine present in human organisms comes from the food supply and from an endogenous synthesis occurring in the liver and the kidney [11]. Once synthesized, L-carnitine is distributed to tissues and organs whose metabolism is dependent on fatty acid metabolism. Muscles concentrate most (up to 95%) of all the L-carnitine present in human organism. Alteration in L-carnitine homeostasis leads to a decrease in muscle function and a deterioration in neuronal functions. This effect is due to a decrease in oxidative pathways, an increase in free radical production and likely to an impairment in other functions depending on L-carnitine. L-carnitine can be regarded as a regulatory nutrient able to control the metabolic flux and that can improve muscle energy production and muscle function.

Previous studies described alterations in L-carnitine metabolism in DMD patients. Berthillier was the first, in 1982, to report muscle carnitine deficiency in 12 children affected with Duchenne muscular dystrophy (DMD) [12]. These results were later confirmed by two other studies [13], [14]. The molecular bases of this decrease in L-carnitine level are not established yet. Several hypotheses may be drawn. Altered structure of the muscle cell, decreased activity in patient muscle, alteration in the exchange through cell membrane, are among the hypotheses that may explain this decrease in L-carnitine content in patient cells. But, whatever the origin is, DMD patients muscles have, clearly, a major defect in L-carnitine homeostasis.

Besides L-carnitine alteration, lipid content and lipid metabolism have been reported altered in Duchenne patient cells coming from different tissues. In erythrocytes harvested from DMD patients, a decrease in the concentration of unsaturated fatty acids (oleic, linoleic and arachidonic acids) and conversely, an increase in saturated fatty acid amounts were observed [15]. Carroll et al. (1983) reported an increase of long chain acyl CoA in muscle from patients with DMD while free and short chain acylcarnitine were reduced, suggesting a disruption of fatty acid oxidation [16]. More recently, a case of Duchenne muscular dystrophy and severe mental retardation was described in a very young boy with chromosomal anomaly, medium chain fatty acid level was found to high in the cerebrospinal fluid [17].

In mdx mice, alteration in phospholipids composition has also been reported [18], [19]. Even et al. (1994) also reported an impaired fatty acid metabolism in mdx mice and interestingly notice that several major symptoms observed in the muscles of mdx mice seems similar to those observed in muscles of patients or animals suffering severe food restriction [20]. The relation between the fatty acid composition and the severity of the disease was studied in dystrophic muscle. Phospholipids extracted from mdx mice contained less docosahexaenoic acid (C22:6 n–3) and more linoleic acid (C18:2 n–6) and some correlation can be drawn between phospholipid composition and muscle strength [21].

The lipid tails of the phospholipids composing the plasma membranes can affect mechanical properties, including its resistance to stretching and bending. Alterations in the lipid metabolism of heart from mdx mice have also been reported [22]. Mdx heart perfusions with stable isotopes revealed a marked shift in substrate fuel selection from fatty acids to carbohydrates, suggesting, again, alterations in fatty acid metabolism. However, none of these studies investigate in depth the biochemical and molecular bases of these changes in DMD patients.

Surprisingly, identifying lipid composition alterations in human muscle cells has never been done. Plasma membranes of DMD patient cells seem to undergo several rearrangements in terms of lipid composition. The fatty acid composition of the phospholipids present in the membranes is a consequence of both the food supply and the lipid metabolism.

Membrane structure has been studied years before the discovery of dystrophin and very little since. In the early 80′s Rowland [23] reported that many changes occurred in DMD muscle cell membrane, like membrane weakness and alterations in calcium metabolism and regarding the origins of such changes this author wrote “The evidence is by no means conclusive, however, and some of it is contradictory”.

In general, the content of long chain fatty acids and polyunsaturated fatty acids in membrane phospholipids influences membrane rigidity and very likely membrane fragility [24], [25]. If fatty acid metabolism is altered, membrane composition and structure are likely to be changed.

Few studies aimed at looking at membrane fluidity in DMD muscle cells were conducted. In 1986, Chabanel et al. reported alteration in membrane elasticity in DMD patient erythrocytes [26]. Membrane fluidity was also studied on intact fibroblasts, erythrocyte ghosts, and intact lymphocytes from DMD patients [27]. These authors found alterations in membrane fluidity but their conclusions were about the implication of a toxic factor which attacks lymphocyte membranes and possibly muscle membranes at the same time. Several other studies showed fluidity changes in DMD cells, most of them on erythrocytes and all of them before 1985. Studying muscle membrane fluidity and integrity with recent approaches could give rise to new information.

The aim of this project was to characterize metabolic alterations occurring in DMD patient muscle cells and the consequences of such changes on membrane composition and the physiological function of these membranes. An eventual protective role of L-carnitine was also estimated. Our objectives were to (i) determine the alterations in lipid composition of human muscle cell membrane in Duchenne patients, (ii) identify the origins of these changes by identifying the metabolic pathways that are altered by the absence of dystrophin, (iii) characterize the physiological consequences of these alterations in terms of membrane structure and fluidity, and (iv) determine if a supplementation in L-carnitine is able to counteract some of the deleterious effects of Duchenne disease on these metabolic parameters.

## Materials and Methods

### 1 – Chemicals, antibodies

All chemicals were purchased from Sigma (St Quentin Fallavier, France). Culture medium, fetal bovine serum and other cell culture ingredients were purchased from Lonza (Levallois, France).

### 2 – Cells and culture

All these experiments were carried out on human cells provided by Myosix and the AFM. Cells were cultured as recommended and differentiated before use. Our experiments were carried out on patient cells MX00709MBS and unaffected MX01809MBS cells. MX00709MBS cells derived from a 14 year-old male DMD patient, they were CD56 positive at 92%. MX01809MBS cells were derived from an unaffected 13 year-old boy and were found to be CD56 positive at 94%. Cells were cultured in HAM-F10 supplemented with 20% FCS, 1% PS, 480 ng/mL of Dexamethasone, and 10 ng/mL of beta-FGF. Cells (80% confluence) were differentiated 72 hours before use by replacing the culture medium by a differentiation medium composed of DMEM, 1% Penicilin/Streptomycin, 1% glutamine, and 2% Horse serum. L-carnitine was prepared as a stock solution (100 mM), filtrated and added to the culture medium at a final concentration of 500 µM.

### 3 – Carnitine determination, carnitine transport activity

The amount of L-carnitine present in the cells was estimated as previously described [28]. L-carnitine transport activity was determined using tritiated L-carnitine (L-[methyl-^3^H]carnitine, specific activity 80 Ci/mmol from Amersham Pharmacia Biotech (Saclay, France) as previously described [29]. Briefly, L-carnitine uptake studies were carried out at 37°C, in 12-well plates with cells at the density of 25×10^4^ per cm^2^. The medium contained 12.5 nM of radiolabeled L-carnitine and unlabeled L-carnitine (100 µM). After a 30-min incubation, the medium was removed and the cells were washed, scraped in 1 mL of phosphate buffer saline and the radioactivity measured.

### 4 – Fatty acid profile determination

Fatty acid profile and cholesterol content of DMD and healthy cells were determined using Gas Chromatography-Mass Spectrometry (GC/MS). Cells were harvested in PBS, pelleted by centrifugation, membranes were prepared by differential centrifugation [30]. Lipids were extracted from the membranes according to [31] and the lipid composition in phospholipids was analyzed. Fatty acid were derived with PFBBr (pentafluorobenzylbromide) and DIPEA (diisopropylethylamine) and analyzed on HP5MS column (Agilent, 30 m×0.25 mm) and a mass detector (Agilent, MSD 5973). The gas used as a vector was Helium.

Sterols were analyzed after lipid extraction (see above) and separated on a GC/MS apparatus with a HP5MS column and a mass detector (both from Agilent).

### 5 – mRNA profile

This study was conducted by RT-Q-PCR. Total RNA was isolated from differentiated muscle cells using the Trizol reagent according to the manufacturer's protocol (Invitrogen, Carlsbad, CA, U.S.A.) and RNA were treated with DNAse I before reverse transcription. Reverse transcription was performed using random primers and the M-MLV reverse transcriptase (Promega, France). Amplification was done for RNA involved in fatty acids, cholesterol, carnitine, energy metabolism and in inflammation. Primers were designed using Primer-blast and Primer Premier (sequences in supplementary data). PCR reactions were conducted with the Mesagreen QPCR reagents (Eurogentec, Belgium) and reactions were conducted in a StepOne apparatus (Applied biosciences). mRNA levels were compared between untreated and L-carnitine treated cells using the REST algorithm [32] using the actin, 18S and RPLP0 as standards.

### 6 – Membrane fluidity

Plasma membrane fluidity of DMD cells cultured in the presence or in the absence of L-carnitine was estimated by fluorescence anisotropy (*r*) measurement using the hydrophobic fluorescent probe 1,6 diphenyl 1,3,5 hexatriene (DPH, Sigma) to label plasma membrane. *r* was measured using the vertically polarized excitation with the horizontal and vertical emission components as shown by Equation 1, where *I* is the intensity, the first subscript is the position of the excitation polarizer and the second the position of the emission one (*H*: horizontal; *V*: vertical) and *G* is the grating factor defined by Equation 2.
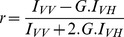
(1)

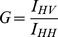
(2)


Cell pellets were harvested after centrifugation at 1000×g for 5 min at 4°C and then resuspended in Opti-MEM® Reduced Serum Medium (Life Technologies, Saint Aubin, France) at a concentration of 10^6^ cells per mL. Prepared samples were subsequently introduced into a 1 cm path length spectroscopic quartz cuvette (VWR International, Limonest, France) placed in a stirred and thermostatically controlled chamber in a Fluorolog®-3 spectrofluorimeter with a T configuration (Jobin-Yvon, Horriba Group, Edison, NJ, USA). After 6 min maintenance at 37°C, plasma membrane was labeled by introducing 2 µL of a stock solution of the fluorescent probe DPH into 2 mL samples. The signal-to-noise ratio which was at least 8 was appreciated through *I_vv_* measurement. The concentration of the stock solution was 1 mM in tetrahydrofuran (Sigma) and it was stored at −20°C in absence of light. *r* was measured after 23.3 min maintenance at 37°C in the presence of DPH then, cell suspensions were chilled to 4°C.

### 7 – Statistical analysis

Statistical analyses were done with a Mann-Whitney test. Significance was assumed at P<0.05. In the tables and figures, two identical letters placed after the values indicated a significant difference between the two samples.

## Results and Discussion

### 1 – Alteration in L-carnitine metabolism in DMD cells

**Figure 1:**
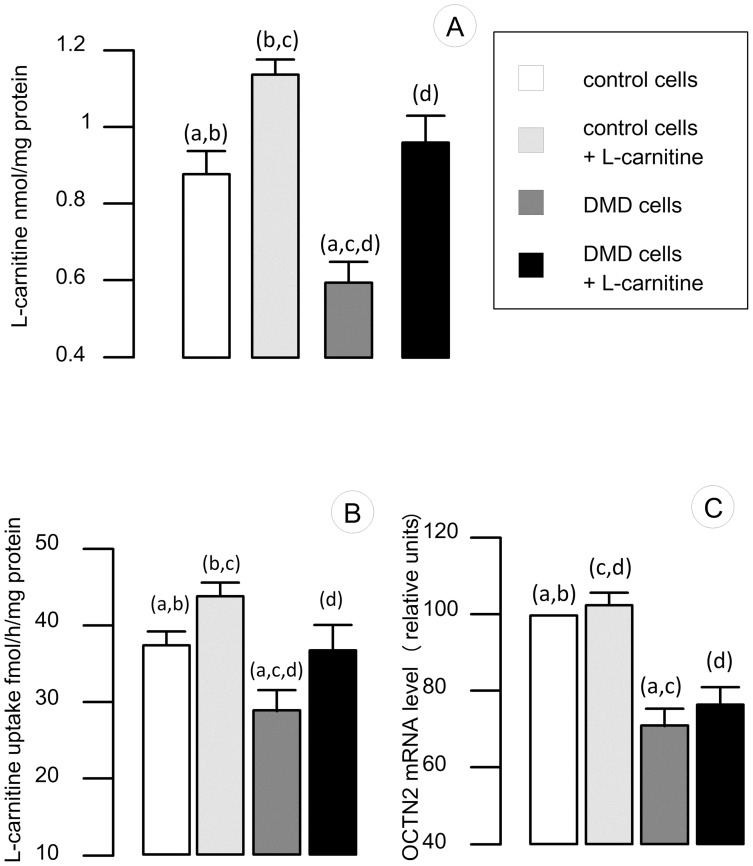
L-carnitine related parameters in muscle cells. L-carnitine content, transport and OCTN2 mRNA levels were determined in control and DMD patient cell treated (or not) with 500 µM of L-carnitine. Results are presented as histograms. Each histogram represented the means +/− sem of 7 independent determinations. Control cells were represented by white histogram, control cells treated with L-carnitine by light grey histogram, DMD cells by dark grey histogram and L-carnitine treated DMD cells by a black histogram. Statistical differences between samples are indicated by letters on top of the histograms. Two identical letters placed indicated a significant difference between the two samples (p<0.05). (**A**) L-carnitine content was determined in cultured muscle cells and L-carnitine content was expressed in nmol per mg of protein. (**B**) L-carnitine uptake was determined in cultured cells and expressed in fmol of L-carnitine transported per hour and per mg of protein. (**C**) OCTN2 mRNA levels were determined by RT-q-PCR. The amount was normalized and expressed relatively to control cells.

As shown in figure 1, a 34% decrease in L-carnitine content was found in differentiated patient cells. This reduced level in L-carnitine was associated with a decrease in the L-carnitine uptake. In patient cells, L-carnitine transport was found to be 23% less than in control cells. In muscle cells, L-carnitine uptake is carried out by OCTN2 [33], the level of OCTN2 mRNA level was estimated by RT-Q-PCR and a 28% decrease in OCTN2 mRNA was found.

Decrease in L-carnitine level is one of the features of Duchenne disease. It has been described in the early 80 s [34] but still remain incompletely understood. Decrease in L-carnitine content and L-carnitine related enzymatic activities observed in Duchenne patients may be the result of a decrease in energy needs in muscle cells, it may also be due to the alteration in membrane structure that may lead to a slower process of fatty acids and carnitine transport through the muscle cell membranes. In the present model, none of the cells are submitted to contraction and the difference in L-carnitine content may then look associated with altered structure of plasma membranes. It is possible to hypothesize that the changes in membrane structure associated with the absence of dystrophin may alter carnitine uptake. Other compounds appear to be abnormally transported into DMD cells: calcium uptake, for instance, has been shown to be dramatically increased in DMD cells [35]. Furthermore, the distribution of several proteins involved in energy production has been shown to be altered, as, for instance for inositol 1,4,5-trisphosphate receptors [36]. OCTN2 is a transmembrane protein whose primary structure is not altered in DMD patient cells but whose function is likely to be altered by the lack of dystrophin and the subsequent membrane rearrangements.

Adding L-carnitine into the culture medium allowed to increase L-carnitine levels in both control and patient cells. In control cells, the increase was +26% and in patient cells a 56% increase in L-carnitine content was observed. The level of L-carnitine in L-carnitine treated patient cells was in the same range than control. Adding L-carnitine allowed to increase L-carnitine uptake by 16% in control cells and by 28% in DMD patient cells. L-carnitine had no significant effect on L-carnitine transport or on OCTN2 mRNA level (figure 1). L-carnitine supplementation allowed to restore L-carnitine levels in DMD cells. This increase in L-carnitine level is not associated with an increase in OCTN2 activity or mRNA levels. L-carnitine supplementation does not modify OCTN2 gene expression but allows for more L-carnitine bioavailable, permitting an increase of intracellular carnitine.

### 2 – Alterations in the lipid composition of the phospholipids of muscle membranes in DMD patients

Lipid (cholesterol and fatty acid) profile was determined in membranes extracted from human cells derived from patients and healthy controls by GC/MS and our data showed that the relative proportion of fatty acids changed between control and patient cells and following L-carnitine treatment (Table 1 and supplementary data).

**Table 1:** Cholesterol content and Fatty acid composition of phospholipids in control, treated and patient muscle cells.

	Control	Control + LC	DMD	DMD + LC
Medium chain fatty acids	4.7±0.6 (a, b)	2.9±0.4	2.8±0.4 (a)	3.3±0.3 (b)
Saturated fatty acids	46.1±5.9 (c)	42.6±6.2 (d)	44.0±2.1 (e)	66.9±3.4 (c,d,e)
Mono-unsaturated fatty acids	47.4±4.6 (f, g)	51.0±2.6 (h, i, j)	41.3±3.3 (f, i, k)	25.6±4.9 (g, j, k)
Poly unsaturated fatty acids	6.5±0.8 (l)	6.3±0.7 (m)	14.6±1.5 (l, m, n)	7.5±1.8 (n)
Very long chain fatty acids (>20)	4.8±1.0 (o)	4.9±0.6 (p)	10.2±1.9 (o, p, q)	4.6±0.8 (q)
Cholesterol	7.89±0.9 (r, s)	8.02±1.1 (t, u)	5.46±0.4 (r, t)	4.95±0.7 (s, u)

Membrane fatty acid profile was determined using GC/MS. For each fatty acid, the relative amount (amount for each FA/total FA amount) was calculated and fatty acids were set in several classes: medium chain fatty acid (from C10 to C14), saturated fatty acid (from C10 to C26), mono- and polyunsaturated fatty acids and finally very long chain fatty acids (C>20). Control cells were cultured under regular conditions (Control) or in the presence of L-carnitine (500 µM), DMD patient cells were also cultured either in the absence (DMD) or the presence of L-carnitine (DMD + LC 500 µM). In the table, each value represents the percentage of the FA family concerned. As many fatty acids can be present in several columns (eg a saturated very long chain fatty acid is going to be present in both the saturated and the VLCFA columns) the total is likely to be different of 100. Cholesterol level is expressed in µg per million cells. Each value is the average + sem of 7 experiments. (letters indicate significant difference P<0.05).

In DMD differentiated muscle cells, some major changes were recorded: the relative amount of VLCFA and PUFA was doubled (x2.1 and x2.2, respectively). The amount of mono unsaturated fatty acids was diminished by 13% in patient cells and the amount of medium chain fatty acids was reduced by 38%. In DMD cells, the amount of cholesterol was diminished by 36%. The relative amount of saturated fatty acids remained the same between control and patient cells. A similar pattern was described in muscles of patients [16].

The concomitant reduction of medium chain fatty acids and the increase in long chain fatty acids has been described as a consequence of a disrupted fatty acid oxidation [37] or, at least, of an altered fatty acid metabolism. Alteration in fatty acid metabolism has been described a long time ago, in DMD patients [38]. In patient, it is always difficult to estimate the major cause of these changes as they may result from the structural and metabolic changes occurring in muscle cells or from the reduced muscle activity due to the effect of the disease. In our cell model, fatty acid profile is not influenced by physical activity and the observed changes are clearly related to alteration in fatty acid metabolism and principally to a decrease in mitochondrial metabolism.

L-carnitine supplementation has very limited effect on control cells (a 38% decrease in MCFA) but major effects on DMD cells. This is not an unexpected result, as in many cases, the supplementation of normal cells has very limited effect [39]. On the other hand, on patient cells, L-carnitine supplementation induces a decrease of 38% of the relative amount of MUFA, a 52% increase in saturated fatty acids, a 49% decrease in PUFA and a 61% decrease in VLCFA. L-carnitine seems to be able to increase mitochondrial activity and partially restore mitochondrial fatty acid oxidation. There is a large amount of publications that describe a regulatory role of L-carnitine on altered fatty acid metabolism (reviewed in [40]).

Cholesterol content was markedly changed in DMD patient cells. Cholesterol levels were reduced by 35% in DMD cells. Cholesterol metabolism has not been extensively studied in DMD patients. In the past, it has been reported changes in cholesterol content in DMD patient cells, in 1983, Fischbeck et al. reported changes in the repartition of cholesterol in patient cells with an overall increase in cholesterol content [41], more recently Tahallah et al. also reported a redistribution of cholesterol in cells lacking dystrophin [42]. Cholesterol (as well as fatty acids, proteins and many other compounds) is an important factor in stabilizing the muscle cell membrane, its content but also its repartition are critical for the stability of the structure of the membrane [43]. The lack of dystrophin has an effect on membrane structure and likely induces a defect in cholesterol inclusion in cell membrane. L-carnitine supplementation had no effect on cholesterol content in muscle membrane neither in patient nor in control cells. This suggests that the defects in cholesterol and fatty acids contents in DMD cells are not directly linked. Restoring L-carnitine level allowed for restoring fatty acid oxidation but remained ineffective for restoring cholesterol content in membranes.

### 3 – Molecular bases of the alterations of the lipid profile in DMD patients cells

Plasma membrane composition as well as intracellular content in lipids is strictly dependent on fatty acid metabolism that was estimated by quantifying mRNAs of key enzymes of these metabolic pathways (Table 2).

**Table 2:** mRNA expression for mitochondrial and peroxisomal metabolisms of fatty acids.

	Control	Control + LC	DMD	DMD + LC
Mitochondrial metabolism				
CPT 1	100 (a, b)	102±11 (c, d)	37±4 (a, c)	42±5 (b, d)
CPT2	100 (e, f)	109±10 (g, h)	29±7 (e, g)	33±7 (f, h)
CACT	100 (i, j)	96±6 (k, l)	33±5 (i, k)	40±7 (j, l)
OCTN1	100 (m, n)	100±11	87±8 (m)	85±7 (n)
ACOT2	100 (o, p)	103±8 (q, r)	43±6 (o, q)	51±5 (p, r)
ACSL1	100 (s, t)	103±8 (u, v)	62±8 (s, u)	62±7 (t, v)
Peroxisomal metabolism				
ACOX1	100 (w, x)	81±7	75±7 (w)	73±7 (x)
EHHADH	100	115±10	87±8	118±10
Thiolase	100	106±10	87±6	91±9
SCP	100 (y, z)	123±8	77±9 (y)	79±6 (z)

mRNA levels were determined on cells after extraction of mRNA and reverse transcription. Results are presented in relative expression of mRNA compared to control cells. Each value is the average + sem of 6 experiments. (Letters indicate significant difference P<0.05). Control cells were cultured under regular conditions (Control) or in the presence of L-carnitine (500 µM), DMD patient cells were also cultured either in the absence (DMD) or the presence of L-carnitine (DMD + LC (500 µM)). Enzymes of the mitochondrial metabolism are CPT1 (Carnitine palmitoyl transferase 1), CPT2 (Carnitine palmitoyl transferase 2), CACT (Carnitine acylcarnitine translocase), OCTN1 (Organic cation transporter new 1), ACOT2 (Acyl-CoA thioesterase 2) and ACSL1 (acyl-CoA synthetase long-chain family member 1, a cytosolic enzyme required for FA activation). Studied peroxisomal enzymes were ACOX 1 (acyl-CoA oxidase 1), EHHADH (enoyl-CoA, hydratase/3-hydroxyacyl CoA dehydrogenase), thiolase (3-Ketoacyl-CoA thiolases) and SCPx (Propanoyl-CoA C-acyltransferase).

Analysis of mitochondrial enzymes showed a marked decrease in the amount of mRNA encoding enzymes involved in the mitochondrial beta oxidation. mRNA levels for CPT1, CPT2, and CACT the three protein of the carnitine shuttle, ACOT2 the mitochondrial thioesterase and the cytosolic enzyme ACSL1, the enzyme involved in the activation of long chain fatty acid into acyl-CoA were significantly reduced (decrease ranging from 71 to 38%). OCTN1 is an L-carnitine transporter that has been described in mitochondrial membranes [44], it does not seem to be involved in the L-carnitine dependent transport of fatty acids across mitochondrial membranes but likely in other mitochondrial functions for L-carnitine. OCTN1 mRNA level was also significantly decreased but to a less extend (−18%). This also strengthened the hypothesis of a reduced mitochondrial metabolism of fatty acid in the mitochondria. Similar data were recently presented in dog lacking dystrophin [45]. In these dogs, an overall decrease in the expression of enzymes involved in energy production was described.

Our data also showed that the levels of mRNA for peroxisomal enzymes remained unchanged in DMD patient cells (Table 2). Peroxisome is not implicated in energy production [46] but primarily in the synthesis of complex fatty acids. The supplementation with L-carnitine did not modify the expression of neither the mitochondrial nor the peroxisomal mRNAs. While L-carnitine has a major impact on fatty acids composition in the cells, this is not associated with changes in mRNA levels and, more likely, L-carnitine supplementation might improve enzymatic and/or transport activity in and across the mitochondria.

All together, these data suggest that dystrophin-lacking cells exhibit a decrease in L-carnitine content associated with changes in the lipid composition and the mitochondrial lipid metabolism. These biochemical changes have impact on the structure of the membrane of the cell as they are associated with changes in the composition of phospholipids and the cholesterol distribution in membrane. L-carnitine supplementation allowed to restore L-carnitine level in muscle cells but it does not modify mRNA levels for mitochondrial enzymes of the beta oxidation.

### 4 - Cell membrane structure estimated by fluorescence anisotropy

**Figure 2:**
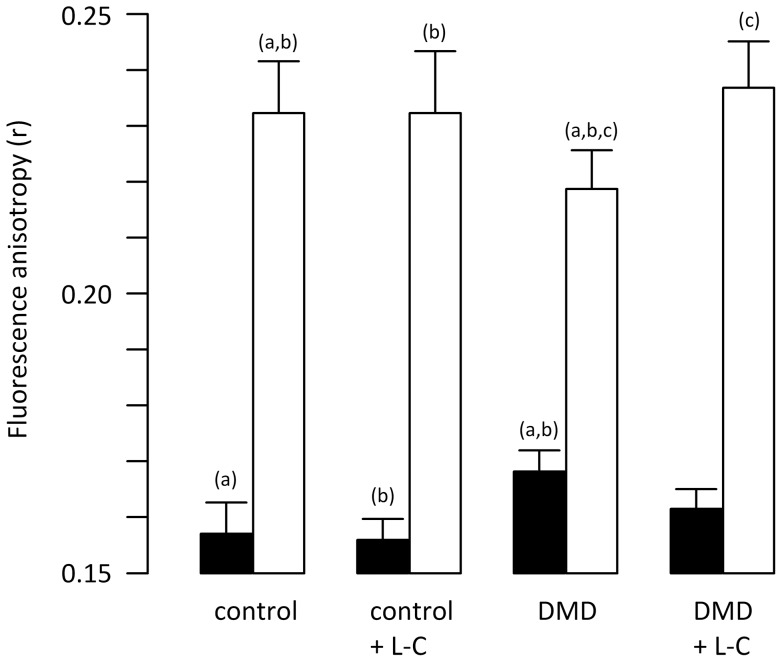
Appreciation of plasma membrane fluidity of normal and patient cells through DPH fluorescence anisotropy measurement (*r)*. Fluorescence anisotropy was measured at (▪) 37°C and (□) 4°C. An Increase in *r* value represented an increase in plasma membrane rigidity and so a decrease in fluidity. The means of at least three independent measurements were calculated and the 95% confidence intervals of the means are presented. Two identical letters placed above the histogram indicated a significant difference between the two samples. Control and DMD cells were either untreated or treated with 500 µM of L-carnitine.

Plasma membrane properties of control and patient cells were estimated by the mean of changes in fluidity at 37°C (growth temperature) and 4°C using the hydrophobic fluorescent probe DPH. Membrane fluidity parameter was chosen because of its involvement in membrane functionalities as membrane protein activity [47]. As expected, results presented in Figure 2 showed that both in normal muscular cells and patient cells (with or without L-carnitine) the steady-state fluorescence anisotropy (*r*) as membrane rigidity (inversely to membrane fluidity), significantly increased as temperature decreased. Indeed, *r* value was lower than 0.168 at 37°C whereas it was higher than 0.218 at 4°C.

Interestingly, at 37°C and in the absence of L-carnitine, *r* value of control muscle cells (0.157) was found to be lower than this of patient cells (0.168). This indicated that plasma membrane of patient cells was more rigid than this of control. This observation strongly suggests that a severe alteration of the structure of the cytoskeleton could affect plasma membrane fluidity. Thus, reduced cytoskeletal integrity by the mean of lack of dystrophin [48] and some membrane glycoproteins [49] induced a rigidification of the plasma membrane. Using TMA-DPH probe, Mora et al. [50] reported contrary results showing that fluidity of isolated membranes was higher than for membranes of intact human keratinocytes. The increase in fluidity was explained by the loss of interactions between membrane and cytoskeleton during membrane isolation. Nevertheless, our results are in agreement with recent reports by Sharif-Naeini et al. [51] and Patel et al. [52] showing an increase in stretch-activated cation channels activity in dystrophic myocytes which correlated with the destabilization of the cortical actin cytoskeleton and consequent alteration of membrane surface tension. According to these authors, disruption of the cytoskeleton is thought to increase the radius of membrane curvature meaning that unaffected cells are able to form indentations with low radius of curvature and not DMD cells. Because liquid-ordered lipids (in a rigid phase) are preferentially located in regions of low membrane curvature [53] and according to our results one can hypothesize that, global plasma membrane rigidity is higher in patient cells than in control cells.

The influence of the membrane composition was also appreciated at 4°C. At this low temperature, a significant difference in membrane rigidity between control and patient cells was observed. Surprisingly, at 4°C the *r* value of patient cells (0.218) was lower than for control cells (0.231), indicating that, at 4°C, plasma membranes were more rigid in control than in patient cells. At 37°C, the phenomenon was inverted, plasma membrane was more rigid in patient than in control cells, confirming that physical properties of plasma membrane of patient cells were severely altered. The origins of such differences are likely related to the presence of altered interactions between plasma membrane and the cytoskeleton as it is well known that plasma membrane properties are influenced by interactions with proteins [54].

In the presence of L-carnitine, whatever the temperature (i.e, 37 or 4°C), *r* value of normal cells (about 0.156 at 37°C and about 0.231 at 4°C) and so plasma membrane fluidity was found to be similar than in its absence. This observation was in agreement with the fact that similar lipid profiles were recorded with and without L-carnitine (Figure 2). Interestingly, in the presence of L-carnitine *r* value of patient cells tended to reach this of normal ones. Indeed, at 37°C in the presence of this molecule *r* value and so rigidity of patient cells decreased to 0.162 whereas this of normal cells was 0.156 and increased to 0.238 whereas this of normal cells was about 0.231. This observation indicated that L-carnitine could be used to partially restore physical properties of plasma membrane.

### 5- Conclusion

Duchenne dystrophy is a genetic disease due to the lack of dystrophin in muscle cells. Dystrophin is a key molecule in maintaining the cell structure by creating links between the membrane and the cytoskeleton. We described here that the lack of functional dystrophin also leads to a decrease in the intracellular level of L-carnitine and anomalies in lipid metabolism with alteration in several mRNA levels for mitochondrial enzymes involved in the fatty acid metabolism and the energy production and by changes in the fatty acid profile of the membrane phospholipids. These changes are associated with changes in membrane fluidity. At the physiological temperature of 37°C, the membrane fluidity is reduced in patient cells, this is likely due to changes both in fatty acid composition and in the overall organization (phospholipids-protein interactions) of the membrane and its interaction with the cytoskeleton. L-carnitine supplementation allowed to restore the metabolic function and restore the membrane fluidity.

## Supporting Information

Table S1 Nucleotide sequences of the primers used for Q-PCR. The sequences are presented 5′–3′ and from left to right the forward and the reverse primer.
(DOCX)

Table S2 Fatty acid profile of the PL extracted from membranes of control and patient cells. The content for each fatty acid was determined and expressed in percent of the total amount of fatty acids. Those values represent the raw data used for making Table 1. Each number is the average of 7 independent experiments ± sem.
(DOCX)
