# Supplementary material for: Exploration of Lipid Metabolism in Relation with Plasma Membrane Properties of Duchenne Muscular Dystrophy Cells: Influence of L-Carnitine
Source: PLoS One. 2012 Nov 27;7(11):e49346. doi: 10.1371/journal.pone.0049346 (PMC3507830; doi:10.1371/journal.pone.0049346)
Supplement: Table S1 — Nucleotide sequences of the primers used for Q-PCR. The sequences are presented 5′–3′ and from left to right the forward and the reverse primer. (DOCX) [file pone.0049346.s001.docx]

**Supplementary Table S1**

Nucleotide sequences of the primers used for Q-PCR.

Mitochondrial metabolism

CPT1: CGGACGGGATTGACCTGCGG and CCAACTGGAGGGGCTTGCCG

CPT 2: GGAGCCTGGATCAAATTTTGGG and AGCCGCTCCTTCCCCTTTTT

CACT : GCAGTGATGATCCGAGCCTT and TTCAGCCTCACAAGTTGGGG

OCTN1: CTGAATCTCCCCGATGGCTG and ACTGCTGGTACAGCTGTGTT

ACOT 2: GGAGGTTTCAACACAGGAGACT and AGGGCAGAGCTGTCTGCTAA

ACSL1: GAGCAATGGTCACTCACCGA and TCCGTGGACAAACACCTGAG

Peroxisomal metabolism

ACOX1: TGATGCGAATGAGTTTCTGC and CCCTCGTTGGAAAAATGCTA

EHHADH : AGGCAGCAAACCAGAGGAGGT and CCAGCCCACATCCAACCCAGC

Thiolase: CCTCCCCCAGCACCAAGAGGT and AGGGCTGAAGATTTGCAGTGGTGT

HSCP2: CCTCCCCCAGCACCAAGAGGT and AGGGCTGAAGATTTGCAGTGGTGT

Controls

RPLP0 : CCCCATTCTATCATCAACGG and GCCTTGACCTTTTCAGCAAG

Actin: CATCCCCCAAAGTTCACAAT and TTCCTGTAACAACGCATCTCA

18 S: CACGCCAGTACAAGATCCCA and TTCACGGAGCTTGTTGTCCA
