# Supplementary material for: Exploration of Lipid Metabolism in Relation with Plasma Membrane Properties of Duchenne Muscular Dystrophy Cells: Influence of L-Carnitine
Source: PLoS One. 2012 Nov 27;7(11):e49346. doi: 10.1371/journal.pone.0049346 (PMC3507830; doi:10.1371/journal.pone.0049346)
Supplement: Table S2 — Fatty acid profile of the PL extracted from membranes of control and patient cells. The content for each fatty acid was determined and expressed in percent of the total amount of fatty acids. Those values represent the raw data used for making Table 1. Each number is the average of 7 independent experiments ± sem. (DOCX) [file pone.0049346.s002.docx]

|  |  |  |  |  |  |  |  |  |  |  |  |  |  |  |  |  |  |  |  |  |  |  |  |  |  |
| --- | --- | --- | --- | --- | --- | --- | --- | --- | --- | --- | --- | --- | --- | --- | --- | --- | --- | --- | --- | --- | --- | --- | --- | --- | --- |

**Supplementary Table S2**. Fatty acid profile of the PL extracted from membranes of control and patient cells.

| Fatty acid | Control | Control +LC | DMD | DMD + LC |
| --- | --- | --- | --- | --- |
| 8 :0 | 0.217 ± 0.02 | 0.046 ± 0.01 | 0.017 ± 0.00 | 0.071 ± 0.00 |
| 10 :0 | 0.202 ± 0.02 | 0.066 ± 0.01 | 0.115 ± 0.02 | 0.159 ± 0.02 |
| 12 :0 | 1.131 ± 0.17 | 0.456 ± 0.24 | 0.844 ± 0.07 | 1.075 ± 0.09 |
| 14 :0 | 3.15 ± 0.26 | 2.335 ± 0.19 | 1.888 ± 0.20 | 2.044 ± 0.31 |
| 16:1 n-9 | 4.47 ± 0.37 | 3.734 ± 0.25 | 2.182 ± 0.29 | 0.826 ± 0.86 |
| 16:1 n-7 | 4.976 ± 0.39 | 5.266 ± 0.40 | 3.52 ± 0.27 | 3.096 ± 0.38 |
| 16:00 | 31.23 ± 2.04 | 30.394 ± 2.62 | 25.581 ± 2.34 | 39.076 ± 4.69 |
| 18:3 n-6 | 0.146 ± 0.01 | 0.158 ± 0.02 | 0.334 ± 0.02 | 0.117 ± 0.01 |
| 18:3 n-3 | 0.122 ± 0.02 | 0.076 ± 0.01 | 0.106 ± 0.01 | 0.046 ± 0.00 |
| 18 :2 | 2.175 ± 0.18 | 1.886 ± 0.23 | 5.043 ± 0.44 | 3.345 ± 0.39 |
| 18 :1 n-9 | 29.258 ± 2.45 | 32.261 ± 3.21 | 29.648 ± 2.59 | 18.023 ± 2.35 |
| 18 :1 n-7 | 8.14 ± 0.56 | 9.237 ± 0.67 | 5.303 ± 0.64 | 3.367 ± 0.35 |
| 18 :0 | 9.954 ± 0.86 | 9.133 ± 0.87 | 15.225 ± 1.85 | 24.155 ± 2.98 |
| 20 :5 | 0.319 ± 0.03 | 0.349 ± 0.04 | 1.008 ± 0.10 | 0.685 ± 0.05 |
| 20 :4 | 2.615 ± 0.21 | 2.698 ± 0.28 | 5.641 ± 0.67 | 2.48 ± 0.268 |
| 20 :3 | 0.441 ± 0.35 | 0.392 ± 0.03 | 0.873 ± 0.07 | 0.339 ± 0.02 |
| 20 :1 | 0.473 ± 0.33 | 0.463 ± 0.03 | 0.565 ± 0.06 | 0.23 ± 0.02 |
| 20 :0 | 0.09 ± 0.00 | 0.062 ± 0.01 | 0.161 ± 0.02 | 0.185 ± 0.02 |
| 22 :6 | 0.244 ± 0.03 | 0.256 ± 0.02 | 0.725 ± 0.06 | 0.228 ± 0.03 |
| 22 :5 | 0.289 ± 0.04 | 0.324 ± 0.02 | 0.692 ± 0.05 | 0.212 ± 0.02 |
| 22 :4 | 0.169 ± 0.02 | 0.173 ± 0.02 | 0.209 ± 0.02 | 0.072 ± 0.00 |
| 22 :1 | 0.051 ± 0.01 | 0.045 ± 0.00 | 0.086 ± 0.08 | 0.027 ± 0.00 |
| 22 :0 | 0.047 ± 0.01 | 0.03 ± 0.00 | 0.088 ± 0.01 | 0.061 ± 0.01 |
| 24 :1 | 0.018 ± 0.00 | 0.017 ± 0.00 | 0.027 ± 0.00 | 0.011 ± 0.00 |
| 24 :0 | 0.058 ± 0.01 | 0.032 ± 0.01 | 0.09 ± 0.00 | 0.054 ± 0.01 |
| 26 :0 | 0.019 ± 0.00 | 0.011 ± 0.00 | 0.029 ± 0.00 | 0.016 ± 0.00 |
